# Microstructural organization of the corpus callosum in young endurance athletes: A global tractography study

**DOI:** 10.3389/fnins.2022.1042426

**Published:** 2022-11-29

**Authors:** Takashi Tarumi, Marina Fukuie, Takayuki Yamabe, Ryota Kimura, David C. Zhu, Keigo Ohyama-Byun, Seiji Maeda, Jun Sugawara

**Affiliations:** ^1^Human Informatics and Interaction Research Institute, National Institute of Advanced Industrial Science and Technology, Tsukuba, Ibaraki, Japan; ^2^Graduate School of Comprehensive Human Sciences, University of Tsukuba, Tsukuba, Ibaraki, Japan; ^3^Institute for Exercise and Environmental Medicine, Texas Health Presbyterian Hospital Dallas, Dallas, TX, United States; ^4^Department of Radiology and Cognitive Imaging Research Center, Michigan State University, East Lansing, MI, United States

**Keywords:** aerobic exercise, corpus callosum, diffusion tensor imaging, tractography, cortical thickness

## Abstract

**Introduction:**

Aerobic exercise training has been shown to improve microstructural organization of the corpus callosum (CC); however, evidence of this topographic effect is limited.

**Purpose:**

To compare the CC microstructural organization between endurance athletes and sedentary adults using a white-matter fiber tractography approach.

**Materials and methods:**

Diffusion tensor imaging (DTI) and T_1_-weighted structural data were collected from 15 male young endurance athletes and 16 age- and sex-matched sedentary adults. DTI data were analyzed with a global probabilistic tractography method based on neighborhood anatomical information. Fractional anisotropy (FA) and mean, radial (RD), and axial diffusivities were measured in the eight CC tracts: rostrum, genu, splenium, and body’s prefrontal, premotor, central, parietal, and temporal tracts. Cortical thickness of the CC tract endpoints and the CC tract length and volume were also measured. Physical activity level was assessed by metabolic equivalents (METs).

**Results:**

The athlete group had an average VO_2_max of 69.5 ± 3.1 ml/kg/min, which is above 90%ile according to the American College of Sports Medicine guideline. Compared with the sedentary group, the athlete group had higher FA in the CC body’s premotor and parietal tracts and the CC splenium. These tracts showed lower RD in the athlete compared with sedentary group. The voxelwise analysis confirmed that the athlete group had higher FA in the CC and other white matter regions than the sedentary group, including the corona radiata, internal capsule, and superior longitudinal fasciculus. Cortical thickness of the CC tract endpoints and the CC tract lengths and volumes were similar between the two groups. Physical activity levels were positively correlated with FA in the CC body’s parietal (*r* = 0.486, *p* = 0.006) and temporal (*r* = 0.425, *p* = 0.017) tracts and the CC splenium (*r* = 0.408, *p* = 0.023).

**Conclusion:**

Young endurance athletes have higher microstructural organization of the CC tracts connected the sensorimotor and visual cortices than the age- and sex-matched sedentary adults.

## Introduction

The corpus callosum (CC) contains ∼200 million axons and is the largest white matter (WM) fiber bundle in the human brain (Aboitiz et al., 1992). This commissural tract connects the left and right cerebrum and plays a central role in the interhemispheric transfer of sensorimotor, visual, and cognitive information (Clarke and Zaidel, 1994; van der Knaap and van der Ham, 2011). The neurobehavioral function of the CC is supported by a topographical organization with the anterior fibers connecting the frontal cortical regions while the middle and posterior fibers are connected to the parietal, temporal, and occipital areas (Huang et al., 2005; Hofer and Frahm, 2006). In living humans, magnetic resonance diffusion tensor imaging (DTI) measures water diffusion within the brain tissues which can be used to visualize WM fiber tracts *via* its tractography and to assess the microstructural organization *via* anisotropic and diffusivity metrics (Le Bihan et al., 2001). Among the DTI metrics, fractional anisotropy (FA) has been most widely used and increases in highly organized WM fiber tracts (Friedrich et al., 2020).

Accumulating evidence suggests that aerobic exercise training and higher cardiorespiratory fitness are associated with improved microstructural organization of the CC (Sexton et al., 2016; Loprinzi et al., 2020; Maleki et al., 2022). Cross-sectional studies have shown that individuals who regularly perform aerobic exercise or have greater cardiorespiratory fitness have higher FA in the CC, regardless of age or pathological conditions (Chaddock-Heyman et al., 2014; Johnson et al., 2016; Ding et al., 2018; Cao et al., 2021; Tarumi et al., 2021a). Consistently, intervention studies have shown that aerobic exercise training and improved cardiorespiratory fitness are associated with increased FA in the CC in both adults (Bashir et al., 2021; Pani et al., 2022) and children (Chaddock-Heyman et al., 2018). Therefore, these findings suggest that aerobic exercise training attenuates age-related disorganization of the CC while promoting the microstructural development in children, and these benefits of aerobic exercise have hypothetically been mediated by an improved myelination process (Feter et al., 2018).

Despite the abundant evidence supporting the benefits of aerobic exercise for the CC, our understanding of the topographic effect is limited because most of the previous studies conducted region-of-interest or voxelwise analysis using a standard template or atlas which does not consider the spatial distributions of WM pathways inside the CC. For example, the body of CC does not have clear anatomical boundaries but does contain WM fibers projecting to various cortical areas (Huang et al., 2005; Hofer and Frahm, 2006). To date, one study showed a positive correlation between cardiorespiratory fitness and FA in the CC tracts connecting to the premotor area (Johnson et al., 2016), but microstructural organization of the other CC tracts remains unknown. Moreover, aerobic exercise training has been associated with greater cortical thickness involved in the sensorimotor and visual function (Tseng et al., 2013b; Tarumi et al., 2021a). However, cortical thickness of the CC tract endpoints has not been investigated. Therefore, understanding the topographic impact of aerobic exercise training on the CC may help us elucidate the neurobiological mechanism by which aerobic exercise improves neurocognitive function in adults and children.

The purpose of the present study was to characterize microstructural organization of the CC tracts in aerobically trained young adults compared with sedentary control participants. To accomplish this, we used a global tractography technique that calculates DTI metrics in a native brain space based on the prior anatomical information defined by the Human Connectome Project dataset (Maffei et al., 2021). Our primary hypothesis was that aerobically trained adults would exhibit higher FA in the CC tracts connecting the sensorimotor, visual, and prefrontal cortical regions. The secondary hypothesis was that higher FA in the CC is associated with the greater cortical thickness of their connected cortical regions. Finally, we explored group differences in FA at a whole-brain level using voxelwise analysis.

## Materials and methods

### Participants

This study included 15 male endurance athletes and 16 sedentary men whose age was between 18 and 23 years old. The athlete group consisted of middle- and long-distance runners recruited from the track-and-field team at the University of Tsukuba. They have been participating in a structured training program comprising 12 sets of ∼60-min exercise per week. The 12 sets were divided into four sets of high-intensity interval running, four sets of moderate-intensity continuous running, and four sets of low-intensity jogging. Besides, they performed two sets of ∼30-min strength exercise per week (e.g., bench press, squat, dead-lift, and snatch). These athletes had a total running distance of 150–170 kilometers in a typical training week and an average training history of ∼7 years starting from middle or high school. In our recent publication (Tarumi et al., 2021b), our athlete participants exhibited profound cardiovascular adaptations, including the increased aortic compliance and eccentric cardiac hypertrophy with the preserved systolic function, which is also known as “athlete’s heart.” The purpose of the present study is to uncover their brain structural characteristics potentially relating to the unique cardiovascular function found in the previous study (Tarumi et al., 2021b).

Sedentary participants were recruited through community-based advertisement at local websites. The inclusion criterion for the sedentary group was no participation in a structured exercise or physical activity program for the last 3 years. Exclusion criteria for both groups included a history of major medical conditions such as cardiovascular, cerebrovascular, kidney, and neurological diseases. Participants who smoked cigarette currently or in the past were excluded. No participants were taking medications. Individuals with any metal in their body which is not safe for MRI were excluded. All experimental procedures and protocols were approved by the Institutional Review Board of the National Institute of Advanced Industrial Science and Technology and performed by the guidelines of the Declaration of Helsinki and Belmont Report. All participants provided written informed consent before participation.

### Study protocol

Participants visited our institute and answered medical history, physical activity, and MRI safety questionnaires. Height and body mass were measured to calculate body mass index (BMI). Blood pressure and heart rate were measured at rest in the seated position (HEM-7130, Omron Corporation, Japan) and were used for screening hypertension. Subsequently, MRI data were collected on a 3-Tesla scanner (Ingenia, Philips Medical Systems, Netherlands) using a 32-channel head coil (dStream Head) for radiofrequency signal reception and a quad body coil for signal transmission. Maximal oxygen uptake (VO_2_max) was measured on a separate day in the athlete group to determine the aerobic fitness level.

### Measurement and analysis

#### Magnetic resonance imaging acquisition and processing

Diffusion tensor imaging data were acquired with the following parameters: field of view (FOV) = 224 mm × 224 mm, number of axial slices = 65 (no gap), voxel resolution = 2 mm × 2 mm× 2 mm, repetition time (TR) = 4,328 ms, echo time (TE) = 94 ms, flip angle = 90°, single-shot spin-echo echo-planar-imaging sequence with a sensitivity encoding (SENSE) factor = 2, and 64 uniformly distributed diffusion gradient orientations with *b*-value = 1,000 s/mm^2^, along with a non-diffusion weighted image (*b*-value = 0 s/mm^2^). Brain structural data were acquired with T_1_-weighted 3D magnetization prepared rapid acquisition with gradient echo (MPRAGE) imaging with the following parameters: FOV = 256 × 256 mm, number of sagittal slices = 176 (no gap), voxel resolution = 1 mm × 1 mm × 1 mm, TR = 7 ms, TE = 3 ms, flip angle = 8°, and a SENSE factor = 2.2. Additionally, T_2_-weighted fluid-attenuated inversion recovery (FLAIR) images were acquired with the following parameters: FOV = 240 mm × 240 mm, number of axial slices = 48 (no gap), slice thickness = 3 mm, in-plane resolution = 1 mm × 1 mm, and TR = 11,000 ms, TE = 125 ms, and inversion time = 2,800 ms.

All MRI data were processed in a blinded fashion using the FreeSurfer image analysis suite (v7.2)^1^ (Fischl, 2012). The cortical reconstruction and volumetric segmentation were performed on the MPRAGE images with the “*recon-all*” command in its default settings, except the inclusion of FLAIR image to improve pial surfaces. The processing included skull stripping, Talairach transforms, subcortical white and gray matter segmentations, spherical atlas registration, cortical percolation using the *Desikan-Killiany* atlas, and measurement of cortical thickness. Individual images were visually inspected for segmentation errors and no manual correction was required.

Diffusion tensor imaging data were processed with the TRActs Constrained by UnderLying Anatomy (TRACULA) program included as part of the FreeSurfer.^2^ TRACULA uses the prior anatomical information obtained from the brain segmentation and automatically reconstructs WM pathways in a native brain space using a global probabilistic tractography method (Yendiki et al., 2011; Maffei et al., 2021). The image processing was performed with a configuration file for cross-sectional study which executed the following procedures: (1) preprocessing, (2) FMRIB Software Library (FSL)’s BEDPOSTX, and (3) WM pathway reconstruction. The preprocessing included eddy-current correction, head motion calculations during DTI scan (Yendiki et al., 2014), intra- and inter-individual image registrations, extraction of tensor metrics, and computation of anatomical priors for WM pathway reconstruction. Head motion during DTI scan was similar between the athlete and sedentary groups (translation: 0.43 ± 0.15 vs. 0.51 ± 0.23 mm, *p* = 0.236; rotation: 0.0035 ± 0.0011 vs. 0.0048 ± 0.0032 degrees, *p* = 0.166). Second, FSL’s BEDPOSTX with default settings was used to fit the ball-and-stick model of diffusion to reconstruct WM pathways from the DTI data (Behrens et al., 2007). Third, probability distributions for the WM pathways were generated by simultaneously fitting the shape of each pathway to the results of the ball-and-stick model and the prior knowledge of WM pathway anatomy obtained from the manually annotated training dataset (Maffei et al., 2021).

**FIGURE 1:**
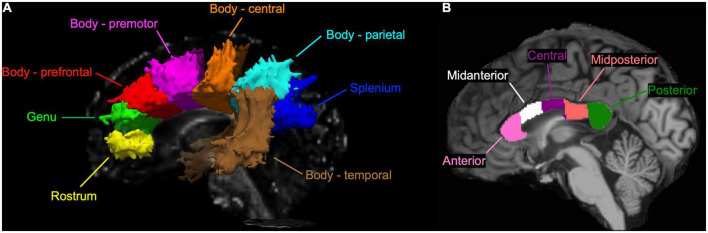
**(A)** A sagittal view of the corpus callosum (CC) tracts reconstructed by the TRActs Constrained by UnderLying Anatomy (TRACULA) in a single participant of the present study. Each tract is displayed at 20% of its peak probability. **(B)** A sagittal view of the CC subsections segmented in T_1_-weighted structural image of the same participant.

With the TRACULA, a total of eight WM tracts were generated in the CC: genu, rostrum, spelinum, and body’s prefrontal, premotor, central, parietal, and temporal tracts according to their endpoint cortical regions. Figure 1A shows the anatomical location of each tract displayed at 20% of the peak probability in a single participant of the present study. Anisotropy and diffusivity measures averaged and weighted over the entire support of path distribution and the average length and volume of each CC tract were computed. In addition, subsection and total volumes of the CC were computed from T_1_-weighted structural image data using the FreeSurfer program (Figure 1B). All volumetric measures were normalized to intracranial volume to control for individual differences in head size. To explore the distribution of significant FA along each tract, we conducted the pointwise assessment of streamline tractography attributes (PASTA) analysis where 1D profile of FA values was generated by averaging consecutive cross-sections of each tract (Jones et al., 2005). Furthermore, to determine whether significant FA voxels are located outside the CC, we performed tract-based spatial statistics (TBSS) where individual FA images were non-linearly registered to the *JHU-ICBM-FA-1 mm* template (Smith et al., 2006; Mori et al., 2008). Subsequently, FA value greater than 0.20 was used as a threshold to remove gray matter and cerebrospinal fluid, and a mean FA skeleton which represents the center of tracts common to all participants was created.

#### Physical activity level

The mode, duration (per day), and frequency (per week) of regular physical activities were asked using a modified Godin Leisure-Time Exercise Questionnaire (Godin and Shephard, 1985). The energy expenditure of each mode of physical activity was estimated from a table of metabolic equivalents (METs) (Ainsworth et al., 2011), and energy expenditures of aerobic exercise at an intensity of ≥3.5 METs (e.g., brisk walking, jogging, and running) were summed up.

#### Cardiorespiratory fitness (maximal oxygen uptake)

In the athlete group, VO_2_max was measured on a treadmill (ORK-7000, Ohtake Root Kogyo, Japan) using a breath-by-breath online gas analyzing system (AE-310s, Minato Medical Science, Japan). During VO_2_max testing, treadmill speed was increased from 8.4 km/hour at an increment of 0.6 km/hour every 60 s until volitional exhaustion (Takayama et al., 2017). VO_2_max was defined by the attainment of the following three criteria: (1) respiratory quotient ≥1.1, (2) ≥90% of the age-predicted maximal HR, and (3) rate of perceived exertion ≥19.

### Statistical analysis

The athlete and sedentary groups were compared by the Mann–Whitney U non-parametric test due to a small sample size. Effect size for group differences was estimated by Cohen’s *d*. Two-way mixed analysis of variance (ANOVA) was used to compare FA of the CC tracts (i.e., group × tract) and cortical thickness of the CC tract endpoint regions (i.e., group × hemisphere) between the athlete and sedentary groups. *Post-hoc* pairwise comparisons for two-way mixed ANOVA were corrected by the Bonferroni method. The Spearman correlations analyzed the associations between continuous variables. Means and standard deviations are reported from the Mann–Whitney U test whereas estimated marginal means and 95% confidence intervals (CI) are reported from the two-way mixed ANOVA. Statistical significance was set *a priori* at *p* < 0.05 for two-sided tests. All statistical analyses described above were performed with SPSS 28 (IBM Inc., Chicago, IL, USA).

Pointwise assessment of streamline tractography attributes was analyzed by a general linear model (GLM) with FA as a dependent variable and group as an independent variable. The GLM analysis was performed by the FreeSurfer statistical analysis tool adapted for 1D data (*mri-glmfit*) and a simulation-based, cluster-wise correction for multiple comparisons (*mri_glmfit-sim*) where a cluster-forming threshold was set at *p* < 0.05 with 5,000 simulations. TBSS was performed with GLM using the “*randomise*” program included in the FSL. Multiple comparisons were corrected by threshold-free cluster enhancement with 5,000 permutations, and the corrected statistical map was thresholded by *p* < 0.05. Anatomical locations of significant FA skeleton voxels were identified by the *ICBM-DTI-81 WM labels* atlas (Mori et al., 2008).

Sample size for this study was estimated based on the results from a previous study that investigated WM microstructural organization in older endurance athletes using TBSS (Tseng et al., 2013a). Based on the results, we expected that sample size of ∼8 in each group would achieve 95% power to detect differences between the athlete and sedentary groups, with the effect size of 1.97 (Cohen’s *d*) and an α-level of <0.05 (two-sided).

## Results

Table 1 shows characteristics of the athlete and sedentary groups. Age and height were similar between the two groups. Body mass and BMI showed a trend of lower values in the athlete group. As expected, athletes had significantly higher METs than sedentary participants, with an average VO_2_max of 69.5 ± 3.1 ml/kg/min which corresponds to >90%ile of men at a similar age group according to the American College of Sports Medicine guideline (American College of Sports Medicine, 2013). At rest, heart rate and diastolic blood pressure were lower in athletes than in sedentary participants. The total brain and intracranial volumes were similar between the two groups.

**TABLE 1:** Characteristics of the athlete and sedentary groups.

Measurements	Athlete	Sedentary	*P*-value
			
	Mean ± SD	Mean ± SD	(MW test)
*n*	15	16	
Age (years)	20 ± 1	21 ± 2	0.250
Height (cm)	171 ± 6	172 ± 6	0.527
Body mass (kg)	58 ± 6	66 ± 12	0.055
Body mass index (kg/m^2^)	20 ± 1	22 ± 4	0.144
Heart rate (bpm)	53 ± 6	66 ± 13	**<0.001**
Systolic blood pressure (mmHg)	109 ± 11	116 ± 12	0.097
Diastolic blood pressure (mmHg)	65 ± 9	72 ± 8	**0.044**
Intracranial volume (cm^3^)	1,482 ± 199	1,536 ± 148	0.304
Total brain volume (%ICV)	79.7 ± 8.3	76.0 ± 7.2	0.220
Physical activity (METs × hours/week)	108 ± 22	13 ± 8	**<0.001**
VO_2_max (ml/kg/min)	69.5 ± 3.1	NA	

Values are means ± standard deviations (SD). *P* < 0.05 are bolded. All participants are male. ICV, intracranial volume; METs, metabolic equivalent; MW, Mann–Whitney U test; NA, not available; VO2max, maximal oxygen uptake.

### Group comparison analysis

**FIGURE 2:**
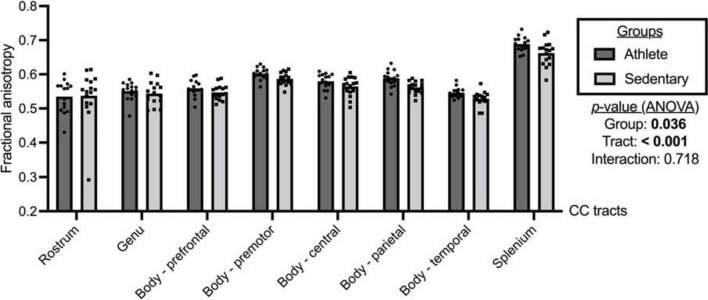
Fractional anisotropy of the corpus callosum (CC) tracts compared between the athlete and sedentary groups. Two-way mixed analysis of variance (ANOVA) examined the main effects of group and CC tract and their interaction effect.

As shown in Figure 2, significant group and tract effects were observed for FA. Specifically, athletes had significantly higher FA across the CC tracts than sedentary participants (athlete vs. sedentary: 0.581, 95% CI 0.571 to 0.591 vs. 0.566, 95% CI 0.557 to 0.576). Comparing FA among the CC tracts (Supplementary Table 1), the splenium (0.676, 95% CI 0.665 to 0.686) exhibited the highest FA followed by the premotor (0.594, 95% CI 0.588 to 0.601), parietal (0.575, 95% CI 0.567 to 0.583), central (0.572, 95% CI 0.563 to 0.581), prefrontal (0.553, 95% CI 0.544 to 0.561), genu (0.547, 95% CI 0.536 to 0.558), temporal (0.537, 95% CI 0.531 to 0.544), and rostrum (0.536, 95% CI 0.513 to 0.559).

**FIGURE 3:**
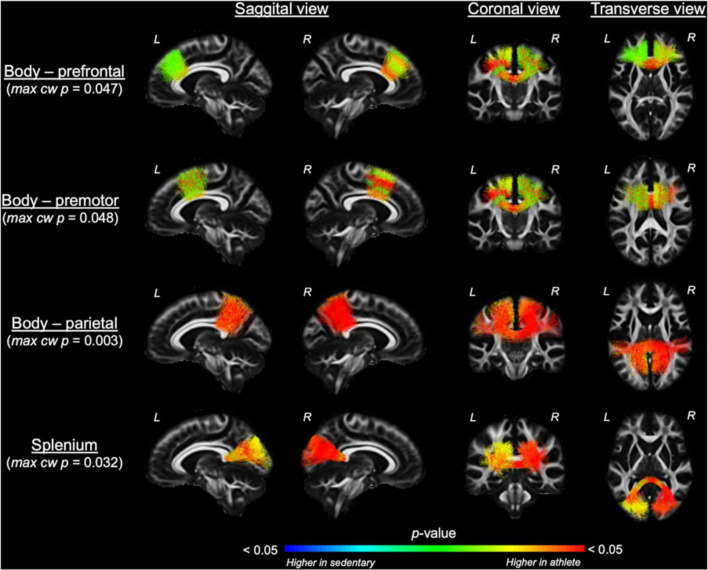
Along-tract fractional anisotropy (FA) analysis of the corpus callosum compared between the athlete and sedentary groups. Results from general linear model analysis were embedded into the template tract files and displayed in the *MGH_HCP_FA_template* brain. The maximal, along-tract cluster-wise *p*-values are presented. L and R denote the **right** and **left** hemispheres.

Comparing each CC tract between the athlete and sedentary groups (Table 2), FA in the body’s premotor, parietal, and temporal tracts and the splenium was significantly higher in the athlete group. Moreover, radial diffusivity (RD) in the body’s prefrontal, premotor, parietal, and temporal tracts and the splenium was significantly lower in the athlete group than the sedentary group. These results were further confirmed by the PASTA showing that FA in the body’s prefrontal, premotor, and parietal tracts and the splenium was significantly higher in the athlete group (Figure 3). Conversely, the lengths and volumes of the CC tracts and subsections were similar between the two groups (Tables 2, 3), except for the splenium showing a significantly shorter length in the athlete group.

**TABLE 2:** Microstructural and morphometric measures of the corpus callosum (CC) tracts computed from the TRActs Constrained by UnderLying Anatomy (TRACULA) in the athlete and sedentary groups.

CC tracts	Measures	Athlete	Sedentary	Effect size	*P*-value
		Mean ± SD	Mean ± SD	(Cohen’s *d*)	(MW test)
Rostrum	FA	0.535 ± 0.047	0.537 ± 0.075	0.040	0.664
	MD	0.769 ± 0.036	0.778 ± 0.045	0.233	0.635
	RD	0.508 ± 0.044	0.512 ± 0.079	0.052	0.664
	AD	1.289 ± 0.067	1.311 ± 0.055	0.362	0.286
	Length	82.0 ± 2.6	82.0 ± 3.4	0.010	0.968
	Volume	0.276 ± 0.115	0.26 ± 0.115	0.135	0.693
Genu	FA	0.551 ± 0.026	0.544 ± 0.034	0.226	0.363
	MD	0.770 ± 0.027	0.774 ± 0.032	0.102	0.813
	RD	0.496 ± 0.028	0.502 ± 0.04	0.190	0.664
	AD	1.320 ± 0.052	1.316 ± 0.049	0.081	0.968
	Length	86.3 ± 2.4	88.2 ± 5.7	0.426	0.664
	Volume	0.353 ± 0.114	0.332 ± 0.121	0.178	0.580
Body–prefrontal	FA	0.559 ± 0.025	0.547 ± 0.022	0.519	0.192
	MD	0.721 ± 0.019	0.736 ± 0.023	0.722	0.105
	RD	0.457 ± 0.025	0.475 ± 0.023	0.737	**0.033**
	AD	1.248 ± 0.036	1.259 ± 0.042	0.266	0.429
	Length	105.3 ± 3.6	105.5 ± 3.3	0.063	0.782
	Volume	0.678 ± 0.149	0.634 ± 0.096	0.351	0.385
Body–premotor	FA	0.602 ± 0.017	0.587 ± 0.018	0.849	**0.030**
	MD	0.713 ± 0.015	0.725 ± 0.015	0.787	0.105
	RD	0.422 ± 0.019	0.438 ± 0.015	1.001	**0.013**
	AD	1.296 ± 0.026	1.298 ± 0.037	0.055	0.693
	Length	103.8 ± 3.9	104.9 ± 3.5	0.290	0.527
	Volume	0.760 ± 0.143	0.721 ± 0.111	0.301	0.477
Body–central	FA	0.580 ± 0.022	0.564 ± 0.028	0.605	0.105
	MD	0.727 ± 0.021	0.735 ± 0.019	0.387	0.252
	RD	0.445 ± 0.024	0.460 ± 0.023	0.608	0.105
	AD	1.291 ± 0.035	1.285 ± 0.045	0.141	0.722
	Length	120.3 ± 3.1	122.3 ± 4.3	0.538	0.144
	Volume	0.865 ± 0.156	0.828 ± 0.144	0.251	0.502
Body–parietal	FA	0.589 ± 0.023	0.561 ± 0.018	1.322	**0.002**
	MD	0.735 ± 0.030	0.757 ± 0.028	0.781	**0.044**
	RD	0.445 ± 0.033	0.476 ± 0.024	1.089	**0.003**
	AD	1.314 ± 0.038	1.320 ± 0.050	0.122	0.635
	Length	134.0 ± 5.1	135.3 ± 5.5	0.232	0.527
	Volume	1.131 ± 0.235	1.075 ± 0.128	0.298	0.843
Body–temporal	FA	0.546 ± 0.015	0.528 ± 0.021	0.965	**0.006**
	MD	0.750 ± 0.021	0.770 ± 0.026	0.817	0.058
	RD	0.487 ± 0.02	0.510 ± 0.027	0.984	**0.011**
	AD	1.278 ± 0.034	1.289 ± 0.041	0.304	0.406
	Length	209.3 ± 9.7	208.8 ± 7.2	0.060	0.968
	Volume	2.039 ± 0.200	1.986 ± 0.146	0.300	0.580
Splenium	FA	0.689 ± 0.022	0.662 ± 0.036	0.889	**0.022**
	MD	0.731 ± 0.027	0.753 ± 0.029	0.788	0.053
	RD	0.373 ± 0.027	0.404 ± 0.039	0.932	**0.011**
	AD	1.448 ± 0.050	1.452 ± 0.060	0.057	0.937
	Length	127.5 ± 3	129.9 ± 4.1	0.679	**0.044**
	Volume	0.699 ± 0.200	0.668 ± 0.110	0.195	0.635

Values are means ± standard deviations (SD). P < 0.05 are bolded. Figure 1A shows the location of each CC tract. MD, RD, and AxD are in × 10^–3^/mm^2^/s while FA is ratio. Lengths are in millimeters and volumes are normalized to intracranial volume and expressed in percentage. AD, axial diffusivity; FA, fractional anisotropy; MD, mean diffusivity; MW, Mann–Whitney U test; RD, radial diffusivity; TRACULA, TRActs Constrained by UnderLying Anatomy.

**TABLE 3:** Subsection and total volumes of the corpus callosum computed from T1-weighted structural image data in the athlete and sedentary groups.

CC subsections	Athlete	Sedentary	Effect size	*P*-value
	Mean ± SD	Mean ± SD	(Cohen’s *d*)	(MW test)
Anterior	0.0617 ± 0.0099	0.0573 ± 0.0072	0.502	0.323
Midanterior	0.0356 ± 0.0085	0.0373 ± 0.0089	0.195	0.752
Central	0.0350 ± 0.0080	0.0346 ± 0.0085	0.054	0.782
Midposterior	0.0333 ± 0.0076	0.0333 ± 0.0046	0.008	0.477
Posterior	0.0630 ± 0.0114	0.0602 ± 0.0058	0.308	0.874
Total	0.229 ± 0.037	0.223 ± 0.026	0.182	0.752

Values are means ± standard deviations (SD). All volumes are normalized to intracranial volume and expressed in percentage. Total volume was calculated as a sum of all the subsections. Figure 1B shows the location of each CC subsection. MW, Mann–Whitney U test.

**FIGURE 4:**
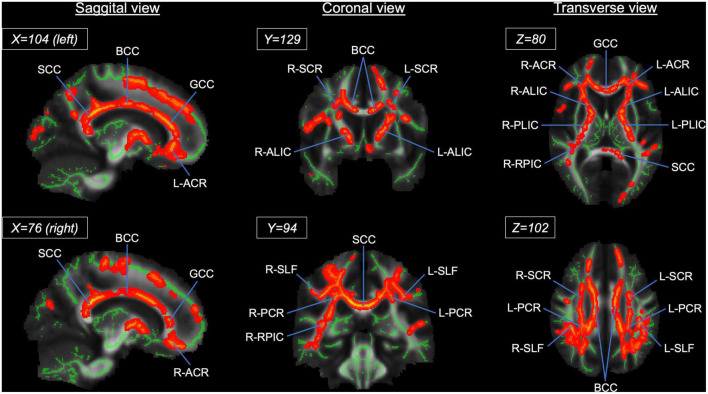
Results from tract-based spatial statistics (TBSS) analysis showing significant white matter skeletons (red) where the athlete group had higher FA than the sedentary group. The coordinates are based on the *JHU ICBM-DTI-81 White-Matter* atlas. Significant skeletons were thickened for visualization. ACR, anterior corona radiata; ALIC, anterior limb of internal capsule; BCC, body of corpus callosum; FA, fractional anisotropy; GCC, genu of corpus callosum; PCR, posterior corona radiata; RPIC, retrolenticular part of internal capsule; SCC, splenium of corpus callosum; SCR, superior corona radiata; SLF, superior longitudinal fasciculus. L and R denote the **right** and **left** hemispheres.

At a whole brain level, TBSS showed that the athlete group had significantly higher FA in the CC and other WM fiber tracts than the sedentary group (Figure 4 and Supplementary Table 2). Specifically, 49.5% of the CC skeleton voxels, including the genu, body, and splenium, exhibited significantly higher FA in the athlete group than the sedentary group. Other WM tracts with significantly elevated FA in the athlete group included the anterior limb and retrolenticular part of internal capsule, the anterior, superior, and posterior parts of corona radiata, and the superior longitudinal fasciculus.

Table 4 shows cortical thickness of the CC tract endpoints in the athlete and sedentary groups. There was no significant group or interaction effect, but significant hemisphere effects were observed. Specifically, cortical thickness of the right-side endpoints was greater for the CC body’s prefrontal and temporal tracts. Conversely, cortical thickness of the left-side endpoints was greater for the CC body’s parietal tract and the splenium.

**TABLE 4:** Cortical thickness of the corpus callosum (CC) tract endpoints compared between the athlete and sedentary groups.

CC tracts	Hemispheres	Athlete	Sedentary	*P*-value (ANOVA)		
		Mean ± SD	Mean ± SD	Group	Hemisphere	Interaction
Rostrum	Left	2.549 ± 0.133	2.535 ± 0.098	0.933	0.457	0.590
	Right	2.523 ± 0.164	2.531 ± 0.111			
Genu	Left	2.571 ± 0.135	2.589 ± 0.094	0.815	0.217	0.631
	Right	2.602 ± 0.146	2.602 ± 0.076			
Body–prefrontal	Left	2.703 ± 0.163	2.740 ± 0.121	0.523	**0.003**	0.793
	Right	2.758 ± 0.195	2.787 ± 0.099			
Body–premotor	Left	2.676 ± 0.144	2.747 ± 0.132	0.537	0.720	**0.001**
	Right	2.72 ± 0.170*	2.711 ± 0.119*			
Body–central	Left	2.375 ± 0.153	2.428 ± 0.149	0.585	0.152	0.371
	Right	2.363 ± 0.216	2.374 ± 0.165			
Body–parietal	Left	2.285 ± 0.135	2.356 ± 0.124	0.293	**0.015**	0.106
	Right	2.273 ± 0.121	2.296 ± 0.134			
Body–temporal	Left	2.652 ± 0.141	2.688 ± 0.082	0.345	**<0.001**	0.804
	Right	2.753 ± 0.156	2.796 ± 0.095			
Splenium	Left	2.098 ± 0.150	2.176 ± 0.109	0.295	**<0.001**	**0.048**
	Right	2.031 ± 0.169*	2.052 ± 0.097*			

Values are mean ± standard deviation (SD) in millimeters. P < 0.05 are bolded. *P < 0.05 vs. left hemisphere. P-values were calculated from two-way mixed analysis of variance (ANOVA).

### Individual correlation analysis

In all participants, significant positive correlations between METs and FA were observed for the CC body’s parietal (*r* = 0.486, *p* = 0.006) and temporal (*r* = 0.425, *p* = 0.017) tracts and the splenium (*r* = 0.408, *p* = 0.023). Higher METs were also correlated with lower RD in the CC body’s parietal (*r* = −0.406, *p* = 0.023) and temporal (*r* = −0.363, *p* = 0.045) tracts and the splenium (*r* = −0.390, *p* = 0.030). No significant correlation between FA and cortical thickness of the corresponding CC tract endpoints was observed (all *p* > 0.05).

## Discussion

The main findings from this study are as follows. First, compared with the sedentary group, endurance athletes exhibited higher FA in the CC, particularly the body’s premotor, parietal, and temporal tracts and the splenium. Higher METs were also correlated with higher FA in these CC tracts in all participants. Second, cortical thickness of the CC tract endpoints was similar between the athlete and sedentary groups and not correlated with FA of the corresponding CC tracts. Third, voxelwise analysis showed that higher FA in the athlete group was not limited only to the CC but also extended to the internal capsule, corona radiata, and superior longitudinal fasciculus. Collectively, these findings suggest that aerobic exercise training is associated with higher microstructural organization of the CC tracts connecting the sensorimotor and visual cortices in young adults.

The CC, the largest WM bundle in the human brain (Aboitiz et al., 1992), exhibits a topographic organization with the anterior fibers connecting the frontal cortical regions and the posterior fibers connecting the parietal, temporal, and occipital cortical regions (Huang et al., 2005; Hofer and Frahm, 2006). Interconnecting the left and right cerebral hemispheres, the CC represents the primary site for the interhemispheric transfer of cognitive and sensorimotor information (Clarke and Zaidel, 1994; van der Knaap and van der Ham, 2011). On the other hand, aerobic exercise training has been shown to associate with higher microstructural organization of the CC tracts regardless of participant ages (Chaddock-Heyman et al., 2014; Ding et al., 2018; Bashir et al., 2021; Cao et al., 2021; Tarumi et al., 2021a; Pani et al., 2022), although its topographic effect has been unclear. Therefore, we used a global tractography method (Maffei et al., 2021) to investigate microstructural organization of the CC tracts in young endurance athletes. To our knowledge, this study provides the first evidence that aerobically trained adults have higher FA in the CC body connecting the sensorimotor and visual cortices when compared with sedentary adults. Overall, these findings may contribute to a better understanding of the neurobiological mechanism by which aerobic exercise improves neurocognitive function.

Endurance athletes showed higher FA in the CC body’s premotor tract than sedentary participants. This finding supports the dogma of “use it or lose it,” which indicates that regular physical exercise may induce adaptations in specific brain regions (Scholz et al., 2009). Accordingly, the higher FA at the premotor WM pathway may facilitate voluntary movement of skeletal muscles and joints during exercise. Our athlete group also had higher FA in the CC body’s parietal and temporal tracts than sedentary adults. With the tractography method used in the current study (Maffei et al., 2021), the parietal tract is connected to the superior parietal lobule, supramarginal gyrus, and precuneus which are responsible for visuospatial orientation directed for motor control and movement (Karnath, 1997; Mahayana et al., 2014). The temporal tract is connected to the temporal gyri and superior temporal sulcus which have a broad range of sensory functions, including auditory and social perceptions (Deen et al., 2015; Yi et al., 2019). The CC splenium is connected to the parietal and occipital cortical regions which are involved in the visuospatial processing of environmental stimuli (Kravitz et al., 2011). Therefore, our findings collectively suggest that aerobic exercise training improves microstructural organization of the CC tracts that are responsible for sensorimotor and visuospatial functions.

Higher FA observed in the CC tracts of endurance athletes was accompanied by lower RD while axial diffusivity was similar between the two groups. Based on the principle of DTI, RD reflects water diffusion perpendicular to the axonal tracts (Le Bihan et al., 2001), and neurobiologically increased RD was shown to be associated with demyelination in the post-mortem (Klawiter et al., 2011) and animal model (Song et al., 2002) studies. Thus, our results suggest that the CC tracts in endurance athletes have greater myelination than sedentary participants, which in turn may increase the conduction velocity of action potentials and facilitate interhemispheric communications. Although this hypothesis is supported by a recent meta-analysis suggesting that physical exercise promotes axonal myelination (Feter et al., 2018), it is important to acknowledge that RD measured by DTI is not specific to myelination (Wheeler-Kingshott and Cercignani, 2009). To determine whether aerobic exercise training promotes myelination, future studies with other imaging techniques, such as myelin water fraction imaging (Alonso-Ortiz et al., 2015), are needed.

In contrast to higher FA observed in the athlete group, cortical thickness of the CC tract endpoint cortical regions was similar between both groups. The dissociation between WM microstructural organization and cortical thickness may be surprising but actually supported by the literature. For example, an age-related decrease in WM integrity inferred by FA was not related to cortical thickness in older adults, and their cognitive performance was associated with the decrease in WM integrity but not cortical thickness (Ziegler et al., 2010). Moreover, our recent study showed that middle-aged endurance athletes had higher global WM FA than sedentary participants, but their cortical thickness exhibited a typical pattern of age-related atrophy (Tarumi et al., 2021a). Consistent with the latter study, the recent meta-analysis and systematic reviews suggest that exercise training, regardless of its modality, does not have a significant impact on cortical thickness or brain volume (Gogniat et al., 2021; Hvid et al., 2021). Therefore, these results from the current and previous studies suggest that WM microstructural organization may be more sensitive to aerobic exercise training than gray matter volume and alter independently from a change in cortical thickness.

The results from TBSS analysis confirmed that the FA in the CC was higher in the athlete compared with the sedentary group, but it also revealed that the athletes had significantly higher FA in the other WM tracts such as the internal capsule, corona radiata, and superior longitudinal fasciculus. Notably, the spatial distribution of significant FA voxels observed in the current study resembled the distributions reported in our previous studies in middle-aged and older adults with normal cognitive function (Tseng et al., 2013a; Tarumi et al., 2021a) and patients with mild cognitive impairment (Ding et al., 2018). Therefore, these findings suggest that aerobic exercise training or having higher cardiorespiratory fitness may improve WM microstructural organization, independently from aging and/or pathological process in the brain.

### Limitations and methodological considerations

This study has several limitations. First, the topographic effect of aerobic exercise training on the CC cannot be inferred from this cross-sectional design and needs to be confirmed by randomized controlled trials. Nevertheless, conducting a randomized intervention study with a prolonged, high-intensity aerobic exercise training program would be daunting, if not impossible. Also, we cannot rule out the possibility that lifestyle (e.g., diet and sleep) and/or genetic factors could have influenced our outcome measures. Second, this study had a small sample size which limited statistical power of detecting significant group differences or correlations, although recruiting highly trained endurance athletes might have helped increase the effect size of aerobic exercise training. Additionally, all participants were men and thus we cannot generalize our findings to women. Because sex differences in WM microstructural organization have been reported in the literature (Kanaan et al., 2014), our results need to be confirmed by a larger sample, including both men and women. Third, although diffusion-weighted MRI is currently the only method capable of assessing WM microstructural organization in living human beings, FA is inherently limited by the anatomical (e.g., crossing fibers), structural (e.g., packing density and diameter), and functional (e.g., membrane permeability) properties of axons (Jones et al., 2013). Therefore, our results need to be interpreted carefully and confirmed by other imaging modalities such as multi-shell diffusion-weighted MRI which can partly resolve issues of intravoxel fiber orientations (Tuch et al., 2002). Fourth, this study did not collect behavioral data. Thus, we could not determine whether differences in the CC microstructural organization were related to cognitive or sensorimotor function. Lastly, VO_2_max data were collected only from athletes to objectively determine their fitness level, but the lack of such data in sedentary participants made us unable to examine the correlation between cardiorespiratory fitness and FA. Our athlete participants also performed resistance exercise included in their training program which could have a confounding effect on the association between aerobic exercise and CC microstructural organization.

## Conclusion

Compared with the sedentary control group, young endurance athletes exhibited higher microstructural organization of the CC tracts connecting the premotor, temporal, and parietal cortical regions, as well as the CC splenium. Conversely, cortical thickness of the CC tract endpoints was similar between the two groups. Therefore, our results suggest that aerobic exercise training may improve an interhemispheric transfer of the sensorimotor and visual information in young adults.

## Data availability statement

The raw data supporting the conclusions of this article will be made available by the authors upon a reasonable request.

## Ethics statement

The studies involving human participants were reviewed and approved by the Institutional Review Board for Human Research at the National Institute of Advanced Industrial Science and Technology. The patients/participants provided their written informed consent to participate in this study.

## Author contributions

TT: conceptualized and administered the study, collected, analyzed, and interpreted the data, drafted and revised the manuscript, and obtained funding. MF: collected and analyzed the data and reviewed the manuscript. TY and RK: collected the data and reviewed the manuscript. DZ: interpreted the data, reviewed and edited the manuscript, and provided supervision. KO-B: administered the study, reviewed the manuscript, and provided supervision. SM and JS: conceptualized the study, reviewed the manuscript, and provided supervision. All authors contributed to the article and approved the submitted version.

## Footnotes

1
https://surfer.nmr.mgh.harvard.edu

2
https://surfer.nmr.mgh.harvard.edu/fswiki/Tracula
