# Supplementary material for: Microstructural organization of the corpus callosum in young endurance athletes: A global tractography study
Source: Front Neurosci. 2022 Nov 29;16:1042426. doi: 10.3389/fnins.2022.1042426 (PMC9745143; doi:10.3389/fnins.2022.1042426)
Supplement: Supplementary file 1 [file Table_1.pdf]

Supplementary Material

Supplementary Table 1: Fractional anisotropy compared among the corpus callosum tracts

Tract A	Tract B	Mean difference (A-B)	95% CI	*p*-value (ANOVA)
Rostrum	Genu	-0.011	(-0.046 to 0.023)	1.000
	Body - prefrontal	-0.017	(-0.056 to 0.022)	1.000
	Body - premotor	-0.058	(-0.100 to -0.017)	**<0.001**
	Body - central	-0.036	(-0.076 to 0.003)	0.103
	Body - parietal	-0.039	(-0.077 to -0.001)	**0.040**
	Body - temporal	-0.001	(-0.038 to 0.035)	1.000
	Splenium	-0.140	(-0.175 to -0.104)	**<0.001**
Genu	Body - prefrontal	-0.006	(-0.024 to 0.012)	1.000
	Body - premotor	-0.047	(-0.066 to -0.029)	**<0.001**
	Body - central	-0.025	(-0.043 to -0.006)	**0.002**
	Body - parietal	-0.028	(-0.048 to -0.008)	**0.001**
	Body - temporal	0.010	(-0.009 to 0.029)	1.000
	Splenium	-0.128	(-0.150 to -0.106)	**<0.001**
Body - prefrontal	Body - premotor	-0.041	(-0.052 to -0.031)	**<0.001**
	Body - central	-0.019	(-0.036 to -0.002)	**0.013**
	Body - parietal	-0.022	(-0.038 to -0.006)	**0.002**
	Body - temporal	0.016	(0.002 to 0.029)	**0.016**
	Splenium	-0.123	(-0.142 to -0.103)	**<0.001**
Body - premotor	Body - central	0.022	(0.008 to 0.036)	**<0.001**
	Body - parietal	0.019	(0.004 to 0.035)	**0.005**
	Body - temporal	0.057	(0.043 to 0.071)	**<0.001**
	Splenium	-0.081	(-0.099 to -0.063)	**<0.001**
Body - central	Body - parietal	-0.003	(-0.021 to 0.015)	1.000
	Body - temporal	0.035	(0.019 to 0.051)	**<0.001**
	Splenium	-0.103	(-0.123 to -0.084)	**<0.001**
Body - parietal	Body - temporal	0.038	(0.028 to 0.0470)	**<0.001**
	Splenium	-0.101	(-0.115 to -0.086)	**<0.001**
Body - temporal	Splenium	-0.138	(-0.152 to -0.124)	**<0.001**

Mean differences, 95% confidence intervals (CI), and *p*-values were calculated from the two-way mixed analysis of variance (ANOVA) shown in Figure 2. All *p*-values are adjusted with the Bonferroni correction.

Supplementary Table 2: Anatomical location of significant fractional anisotropy voxels shown in Figure 4

Region of interest	Hemisphere	Voxel count	(%)
Whole brain		20541	(17.7)
Commissural fibers			
Genu of corpus callosum (GCC)		918	(51.5)
Body of corpus callosum (BCC)		1462	(46.3)
Splenium of corpus callosum (SCC)		1216	(52.3)
Tapetum (Tp)	Left	0	(0)
	Right	0	(0)
Projection fibers			
Anterior corona radiata (ACR)	Left	849	(54.8)
	Right	968	(61.1)
Superior corona radiata (SCR)	Left	670	(47.9)
	Right	874	(59.4)
Posterior corona radiata (PCR)	Left	401	(66.6)
	Right	566	(74.2)
Anterior limb of internal capsule (ALIC)	Left	370	(49.3)
	Right	325	(41.0)
Posterior limb of internal capsule (PLIC)	Left	401	(43.0)
	Right	196	(21.6)
Retrolenticular part of internal capsule (RPIC)	Left	28	(3.8)
	Right	271	(36.1)
Posterior thalamic radiation (PTR)	Left	91	(9.8)
	Right	203	(20.9)
Cerebral peduncle (CP)	Left	29	(5.1)
	Right	0	(0)
Association fibers			
Superior longitudinal fasciculus (SLF)	Left	351	(20.0)
	Right	425	(24.0)
Superior fronto-occipital fasciculus (SFOF)	Left	71	(68.9)
	Right	53	(49.5)
Uncinate fasciculus (UF)	Left	0	(0)
	Right	183	(36.4)
Sagittal stratum (SS)	Left	10	(1.6)
	Right	219	(35.8)
External capsule (EC)	Left	25	(2.8)
	Right	148	(17.1)
Cingulum - cingulate gyrus (CgC)	Left	7	(3.3)

	Right	14	(7.1)
Cingulum - hippocampus (CgH)	Left	0	(0)
	Right	0	(0)
Fornix (cres)/Stria terminalis (FX/ST)	Left	0	(0)
	Right	0	(0)
Brainstem fibers			
Corticospinal tract (CST)	Left	0	(0)
	Right	0	(0)
Superior cerebellar peduncle (SCP)	Left	0	(0)
	Right	0	(0)
Middle cerebellar peduncle (MCP)		0	(0)
Inferior cerebellar peduncle (ICP)	Left	0	(0)
	Right	0	(0)
Medial lemniscus (ML)	Left	0	(0)
	Right	0	(0)
Pontine crossing tract (PCT)		0	(0)

Percentage represents the proportion of significant voxels relative to the whole region. Each voxel is 1 mm^3.
